# Advancing translational research with the Semantic Web

**DOI:** 10.1186/1471-2105-8-S3-S2

**Published:** 2007-05-09

**Authors:** Alan Ruttenberg, Tim Clark, William Bug, Matthias Samwald, Olivier Bodenreider, Helen Chen, Donald Doherty, Kerstin Forsberg, Yong Gao, Vipul Kashyap, June Kinoshita, Joanne Luciano, M Scott Marshall, Chimezie Ogbuji, Jonathan Rees, Susie Stephens, Gwendolyn T Wong, Elizabeth Wu, Davide Zaccagnini, Tonya Hongsermeier, Eric Neumann, Ivan Herman, Kei-Hoi Cheung

**Affiliations:** 1 Millennium Pharmaceuticals, Cambridge, MA, USA; 2 Initiative in Innovative Computing, Harvard University, Cambridge, MA, USA; 3 Laboratory for Bioimaging and Anatomical Informatics, Department of Neurobiology and Anatomy, Drexel University College of Medicine, Philadelphia, PA, USA; 4 Section on Medical Expert and Knowledge-Based Systems, Medical University of Vienna, Vienna, Austria; 5 National Library of Medicine, Bethesda, MD, USA; 6 Agfa Healthcare, Waterloo, Ontario, Canada; 7 Brainstage Research, Pittsburgh, PA, USA; 8 AstraZeneca, Mölndal, Sweden; 9 MassGeneral Institute for Neurodegenerative Disease, Massachusetts General Hospital, Charlestown, MA, USA; 10 Partners HealthCare System, Wellesley, MA, USA; 11 Alzheimer Research Forum, Boston, MA, USA; 12 Harvard Medical School, Boston, MA, USA; 13 Integrative Bioinformatics Unit, University of Amsterdam, Amsterdam, The Netherlands; 14 Cleveland Clinic Foundation, Cleveland, OH, USA; 15 Science Commons, Cambridge, MA, USA; 16 Oracle, Burlington, MA, USA; 17 Language & Computing, Reston, VA, USA; 18 Teranode Corporation, Seattle, WA, USA; 19 World Wide Web Consortium (W3C); 20 Center for Medical Informatics, Yale University School of Medicine, New Haven, CT, USA

## Abstract

**Background:**

A fundamental goal of the U.S. National Institute of Health (NIH) "Roadmap" is to strengthen *Translational Research*, defined as the movement of discoveries in basic research to application at the clinical level. A significant barrier to translational research is the lack of uniformly structured data across related biomedical domains. The Semantic Web is an extension of the current Web that enables navigation and meaningful use of digital resources by automatic processes. It is based on common formats that support aggregation and integration of data drawn from diverse sources. A variety of technologies have been built on this foundation that, together, support identifying, representing, and reasoning across a wide range of biomedical data. The Semantic Web Health Care and Life Sciences Interest Group (HCLSIG), set up within the framework of the World Wide Web Consortium, was launched to explore the application of these technologies in a variety of areas. Subgroups focus on making biomedical data available in RDF, working with biomedical ontologies, prototyping clinical decision support systems, working on drug safety and efficacy communication, and supporting disease researchers navigating and annotating the large amount of potentially relevant literature.

**Results:**

We present a scenario that shows the value of the information environment the Semantic Web can support for aiding neuroscience researchers. We then report on several projects by members of the HCLSIG, in the process illustrating the range of Semantic Web technologies that have applications in areas of biomedicine.

**Conclusion:**

Semantic Web technologies present both promise and challenges. Current tools and standards are already adequate to implement components of the bench-to-bedside vision. On the other hand, these technologies are young. Gaps in standards and implementations still exist and adoption is limited by typical problems with early technology, such as the need for a critical mass of practitioners and installed base, and growing pains as the technology is scaled up. Still, the potential of interoperable knowledge sources for biomedicine, at the scale of the World Wide Web, merits continued work.

## Background

### Translational research and the information ecosystem

Starting in 2002, the NIH began a process of charting a "roadmap" for medical research in the 21st century [1], identifying gaps and opportunities in biomedical research that crossed the boundaries of then extant research institutions. A key initiative that came out of this review is a move to strengthen *Translational Research*, defined as the movement of discoveries in basic research (the *Bench*) to application at the clinical level (the *Bedside*).

Much of the ability of biomedical researchers and health care practitioners to work together – exchanging ideas, information, and knowledge across organizational, governance, socio-cultural, political, and national boundaries – is mediated by the Internet and its ever-increasing digital resources. These resources include scientific literature, experimental data, summaries of knowledge of gene products, diseases, and compounds, and informal scientific discourse and commentary in a variety of forums. Together this information comprises the scientific "information ecosystem" [2]. Despite the revolution of the Web, the structure of this information, as evidenced by a large number of heterogeneous data formats, continues to reflect a high degree of idiosyncratic domain specialization, lack of schematization, and schema mismatch.

The lack of uniformly structured data affects many areas of biomedical research, including drug discovery, systems biology, and individualized medicine, all of which rely heavily on integrating and interpreting data sets produced by different experimental methods at different levels of granularity. Complicating matters is that advances in instrumentation and data acquisition technologies, such as high-throughput genotyping, DNA microarrays, protein arrays, mass spectrometry, and high-volume anonymized clinical research and patient data are resulting in an exponential growth of healthcare as well as life science data. This data has been provided in numerous disconnected databases – sometimes referred to as data silos. It has become increasingly difficult to even discover these databases, let alone characterize them.

Together, these aspects of the current information ecosystem work against the interdisciplinary knowledge transfer needed to improve the bench-to-bedside process.

### Curing and preventing disease requires a synthesis of understanding across disciplines

In applying research to cure and prevent diseases, an integrated understanding across subspecialties becomes essential. Consider the study of neurodegenerative diseases such as Parkinson's Disease (PD), Alzheimer's Disease (AD), Huntington's Disease (HD), Amyotrophic Lateral Sclerosis (ALS), and others. Research on these diseases spans the disciplines of psychiatry, neurology, microscopic anatomy, neuronal physiology, biochemistry, genetics, molecular biology, and bioinformatics.

As an example, AD affects four million people in the U.S. population and causes great suffering and incurs enormous healthcare costs. Yet there is still no agreement on exactly how it is caused, or where best to intervene to treat it or prevent it. The Alzheimer Research Forum records more than twenty seven significant hypotheses [3] related to aspects of the etiology of AD, most of them combining supporting data and interpretations from multiple biomedical specialist areas.

One recent hypothesis on the cause of AD [4] illustrates the typical situation. The hypothesis combines data from research in mouse genetics, cell biology, animal neuropsychology, protein biochemistry, neuropathology, and other areas. Though commensurate with the "ADDL hypothesis" of AD etiology [5], essential claims in Lesné *et al*. conflict with those in other equally well-supported hypotheses, such as the amyloid cascade [6] and alternative amyloid cascade [7].

Consider also HD an inherited neurodegenerative disease. Although its genetic basis is relatively simple and it has been a model for autosomal dominant neurogenetic disorders for many years, [8], the mechanisms by which the disorder causes pathology are still not understood. In the case of PD, despite its having been studied for many decades, there are profound difficulties with some of the existing treatments [9,10], and novel or modified treatments are still being developed [11,12].

Increasingly, researchers recognize that Ad, PD, and HD share various features at the clinical [13], neural [14-17], cellular [18-20], and molecular levels [21,22]. Nonetheless, it is still common for biologists in different subspecialties to be unaware of the key literature in one another's domain.

These observations lead us to a variety of desiderata for the information environment that can support such synthesis. It should take advantage of the Web's ability to enable dissemination of and access to vast amounts of information. Queries need to be made across experimental data regardless of the community in which it originates. Making cross-disease connections and combining knowledge from the molecular to the clinical level has to be practical in order to enable cross-disciplinary projects. Both well-structured standardized representation of data as well as linking and discovery of convergent and divergent interpretations of it must be supported in order to support activities of scientists and clinicians. Finally, the elements of this information environment should be linked to both the current and evolving scientific publication process and culture.

### The Semantic Web

The Semantic Web [23,24] is an extension of the current Web that enables navigation and meaningful use of digital resources by automatic processes. It is based on common formats that support aggregation and integration of data drawn from diverse sources.

Currently, links on Web pages are uncharacterized. There is no explicit information that tells a machine that the mRNA described by <ahref="/entrez/viewer.fcgi?val=NM_000546.2"> on the Entrez page about Human TP53 gene [25] is related to TP53 in any specific way. By contrast, on the Semantic Web, the relationship between the gene and the transcribed mRNA product would be captured in a statement that identifies the two entities and the type of the relationship between them. Such statements are called "triples" because they consist of three parts – *subject*, *predicate*, and *object*. In this case we might say that the subject is *human TP53 gene*, the predicate (or relationship) *hasGeneProduct*, and the object *human TP53 MRNA*. Just as the subject and object – the pages describing the gene and mRNA – are identified by Uniform Resource Identifiers (URIs) [26], so, too, is the relationship, the full name of which might be http://www.ncbi.nlm.nih.gov/entrez/hasGeneProduct. A Web browser viewing that location might show the human readable definition of the relationship.

Since URIs can be used to describe names, all information accessible on the Web today can be part of statements in the Semantic Web. If two statements refer to identical URIs, this means that their subjects of discourse are identical. This makes it possible to merge data references. This process is the basis of data and knowledge integration on the Semantic Web.

With this as a foundation, a number of existing approaches for organizing knowledge are being adapted for use on the Semantic Web. Among these are thesauri, ontologies, rule systems, frame based representation systems, and various other forms of knowledge representation. Together, the uniform naming of elements of discourse by URIs, the shared standards and technologies around these methods of organization, and the growing set of shared practices in using those, are known as Semantic Web technologies.

The formal definition of relations among Web resources is at the basis of the Semantic Web. Resource Description Framework (RDF) [27], is one of the fundamental building blocks of the Semantic Web, and gives a formal specification for the syntax and semantics of statements (triples). Beyond RDF, a number of additional building blocks are necessary to achieve the Semantic Web vision.

• The specification of a query language, SPARQL [28], by which one can retrieve answers from a body of statements.

• Languages to define the controlled vocabularies and ontologies that aid interoperability; the RDF Schema (RDFS) [29], Simple Knowledge Organization System (SKOS) [30], and the Web Ontology Language (OWL) [31].

• Tools and strategies to extract or translate from non-RDF data sources to enable their interoperability with data organized as statements. For example, GRDDL (Gleaning Resource Descriptions from Dialects of Languages) [32] defines a way of associating XML with a transformation that turns it into RDF. There are also a variety of RDF extraction tools and interfaces to traditional databases [33].

Specifications of some of these technologies have published and are stable, while others are still under development. RDF and OWL are about three years old, a long time on the Web scale, but not such a long time for the development of good tools and general acceptance by the technical community. Other technology specifications (SKOS, GRDDL, SPARQL, etc.) will only be published as standards in the coming years – though usable implementations already exist.

Despite the youth of these technologies, active developer and scientific communities have developed around these technologies e.g. SemWebCentral [34]. Today, there are a large number of tools, programming environments, specialized databases, etc (see, e.g., [35]). These tools are offered both by the open source community and as products offered by small businesses and large corporations. Today, we are at the point at which anybody can start developing applications for the Semantic Web because the necessary development tools are now at our disposal.

### How can the Semantic Web help biomedical research?

We have come to believe the judicious application of Semantic Web technologies can lead to faster movement of innovation from research laboratory to clinic or hospital. The Semantic Web approach offers an expanding mix of standards, technologies, and social practices layered on top of the most successful information dissemination and sharing apparatus in existence – the World Wide Web. Some of the elements of the technology most relevant to biomedical research include:

The **global scope of identifiers **that follow from the use of URIs offer a path out of the complexities caused by the proliferation of local identifiers for entities of biomedical interest. Too much effort has been spent developing services mapping between, for instance, the gene identifiers used by the many data sources recording information about them.

The Semantic Web schema languages, RDFS and OWL, offer the potential to simplify the management and comprehension of a complicated and rapidly evolving set of relationships that we need to record among the data describing the products of the life and medical sciences. Along with the benefits of the technologies that underlie our current data stores, there are a number of significant disadvantages that the Web schema languages remediate.

RDFS and OWL are **self-descriptive**. Scientists that integrate different types of data need to understand both what the data means at the domain level, as well as the details of its form as described in associated data schemas. Because these schemas tend to be technology and vendor specific, it is a significant burden to understand and work with them. While the need to integrate more types of data will continue, RDFS and OWL offer some relief to the burden of understanding data schemas. On the Semantic Web, classes and relationships are represented in the same way as the data. Documentation about them is uniformly discoverable due to the standardized *rdf:comment *property. In a well-designed ontology, the structure itself can often help guide users towards its correct use. Some examples of such structure are the well defined meaning of the hierarchical subclass relations, the use of properties defined by the ontology in the construction of definitions within the ontology, and a carefully designed modularization [36].

RDFS and OWL are **flexible, extendable, and decentralized **because they are designed for use in the dynamic, global environment of the Web. RDFS and OWL support hierarchical relationships at their core, allowing for easy incorporation of subclass and subproperty relationships that are essential for managing and integrating complex data. New schemas can easily incorporate previously defined classes and properties that refer to data elsewhere on the Web without the all-too-typical copying and local warehousing of data to be built upon. When different schemas are found to have classes or properties that describe the same kinds of data or relationships, statements may be added that formally record that they should be considered the same. This allows for simpler queries that do not have to account for those equivalences.

The ability to easily extend the work of others makes worthwhile the development of ontologies that can be shared across different domains. For example, there are recent efforts to develop middle ontologies, such as EXPO [37] and the Ontology for Biomedical Investigations (OBI) [38], that are designed to model scientific experiments and investigations. Data from projects that build upon them will be easier to link together than those that use ad-hoc solutions or choose from a variety of disparate and sometimes proprietary LIMS (Laboratory Information Management Software) systems.

Reasoners for the Semantic Web schema languages introduce capabilities previously not widely available by offering the ability to do **inference, classification, and consistency checking**. Each of these capabilities has benefits across the health care and life science domains. For example, the powerful consistency checking offered by OWL reasoners can help ensure that schemas, ontologies, and data sets do not contain contradictory or malformed statements. These erroneous statements are unfortunately quite common. For example, in ongoing work merging two E. coli metabolic databases, 120 cross reference errors were found when comparing descriptions of several hundred metabolites described in both [39]. In a review of Gene Ontology (GO) term usage, up to 10% of terms used for gene annotations were obsolete [40]. When present in research data such errors can lead to missed opportunities. When present in medical records they can result in inappropriate diagnosis and treatment.

We envision the use of Semantic Web technologies will improve the productivity of research, help raise the quality of health care, and enable scientists to formulate new hypotheses inspiring research based on clinical experiences. To help realize this vision, the World Wide Web Consortium (W3C) established the Semantic Web Health Care and Life Sciences Interest Group (HCLSIG) [41] which is chartered to explore and support the use of Semantic Web technologies to improve collaboration, research and development, and innovation in the information ecosystem of the health care and life science domains.

In the remainder of this paper we will describe the makeup and activities of HCLSIG, present a motivating scenario, describe efforts and issues encountered as we have explored the use of Semantic Web technologies, and discuss challenges to and prospects for the approach.

## Methods

### The HCLSIG

The HCLSIG is intended to serve as a bridge connecting the Semantic Web community's technology and expertise to the information challenges and experiences in the health care and life science communities. It pulls together scientists, medical researchers, science writers, and informaticians working on new approaches to support biomedical research. Current participants come from academia, government, non-profit organizations, as well as healthcare, pharmaceuticals, and industry vendors. The ultimate goal is that collaboration between all four groups will help facilitate the development of future standards and tools. Indeed, one objective of a Semantic Web will be to support the effective interaction between academia and industry.

The HCLSIG's role in the effort to create the bench-to-bedside model is to experiment with the application of such standards-based semantic technologies in working with biomedical knowledge. A primary goal is to enable the dynamic "recombining of data", while preserving the layers of meaning contributed by all the participating research groups.

The group's scope is for two years, continuing through the end of 2007. It was chartered with three specific objectives in the domain of Health Care and Life Sciences.

• Identification of core vocabularies and ontologies to support effective access to knowledge and data.

• Development of guidelines and best practices for unambiguously identifying resources such as medical documents and biological entities.

• Development of proposals and strategies for directly and uniformly linking to the information discussed in scientific publications from within those publications – for example the data, protocols, and algorithms used in the research.

The HCLSIG adopts a community-based approach to fostering discussions, exchanging ideas, and developing use cases. It also facilitates collaboration among individual members. In addition to using a public mailing list (public-semweb-lifesci@w3.org) to broadcast and exchange email messages, the HCLSIG conducts regular teleconference calls for members to participate. Wiki pages have been created [42] for describing the various activities in progress within HCLSIG, sharing data and documents produced by individual projects and writing documentation in a collaborative fashion. Face-to-face meetings took place in the United States and The Netherlands to engage the HCLSIG members in closer and more personal interactions as well as working sessions. As a result of the activities from the face-to-face meeting in January 2006, five task forces were established. Each task force plans its work within the two year overall timeframe. The task forces independently, and sometimes collectively, work on different aspects of the overall challenge. These task forces and their goals are described below.

#### BioRDF

Existing biomedical data is available in different (non-Semantic-Web) formats including structured flat files, HTML, XML and relational databases. Often these formats include elements or fields, which are natural language. BioRDF has the goal of converting a number of publicly available life sciences data sources into RDF and OWL. Heterogeneous data sources have been selected so that the group can explore the use of a variety data conversion tools, thereby gaining insight into the pros and cons of different approaches.

#### Ontologies

A goal of the HCLSIG is to facilitate creation, evaluation and maintenance of core vocabularies and ontologies to support cross-community data integration and collaborative efforts. Although there has been substantial effort in recent years to tackle these problems, the methodology, tools, and strategies are not widely known to biomedical researchers. The role of the ontologies task force is to work on well-defined use cases, supporting the other HCLSIG working groups. Where possible, the group works to identify ontologies that formalize and make explicit the key concepts and relationships that are central to those use cases. In cases where ontologies do not currently exist, the group works on prototyping and encouraging further development of the necessary terminology.

#### Drug safety and efficacy

The development of safe and efficacious drugs rests on the proper and timely utilization of diverse information sets and the adoption of and compliance to well-defined policies. The group works on the evaluation of Semantic Web technologies in a number of areas, focusing on the use of ontologies to aid queries against the different information sets, and rules for specification of policies. Topics include:

• Identifying and addressing challenges working with biomarkers and pharmacogenomics in coordination with U.S. Food and Drug Administration (FDA) and European Medicine Agency (EMEA) guidelines.

• Detecting, examining, and classifying signals of potential drug side-effects or adverse reactions [43,44].

• Issues in clinical trial planning, management, analysis, and reporting – e.g., data security and integrity.

• Facilitating electronic submissions as per the Common Technical Document [45] specifications.

#### Adaptable clinical pathways and protocols (ACPP)

Evidence based clinical guidelines and protocols are recommendations for diagnostic and therapeutic tasks in a health care setting. They are increasingly perceived as an important vehicle for moving results of research and clinical trials to application in patient care. Much effort has been devoted to representing clinical guidelines and protocols in a machine-executable format [46]. This has proven to be quite a challenge. Translating the text-based guidelines to a machine-executable format is costly and thus far, solutions have required proprietary guideline execution engines, limiting widespread adoption. The slow pace of updating such guidelines limit their use in medical practices that want to quickly incorporate new clinical knowledge as it is published.

The ACPP task force explores the use of Semantic Web technologies, including RDF, OWL, logic programming, and rules to represent clinical guidelines and guide their local adaptation and execution. Guidelines encoded using these technologies can be accessed, reasoned about, and acted upon by a clinical information system. Since guidelines are Web documents, they have the potential to be more rapidly updated.

The following aspects of guideline and protocol representation and reasoning are of special interest:

• Inclusion and exclusion criteria that are used to decide whether evidence suggests the use of a particular guideline or protocol.

• Representation of temporal concepts and inference rules necessary for tracking processes and ensuring temporal constraints on treatment.

• Representation of medical intentions, goals, and outcomes.

• Use of logic programming to implement guidelines adaptable to site of care execution constraints and changes in patient condition.

#### Scientific publishing

Today, a large portion of biomedical knowledge production is in the form of scientific publications. Most often, on the Web, these publications are referred to either by name or by using hyperlinks. Neither the relationship of the publication to the context from which it is cited, nor the entities and relationships described by it, are explicitly represented. The scientific publishing task force is involved in several activities aimed at ameliorating this situation, attentive to the importance of social process and community engagement.

• Developing an application enabling researchers to collect publications, annotate, and interrelate the hypotheses and claims they present, and share their collections.

• Applying natural language processing techniques to scientific text to recognize and encode entities and relationships among them.

• Creating prototypes of tools and processes to enable researchers to include such information as a standard part of the scientific publication process.

### Neuromedicine and the semantic web

From the outset, HCLSIG participants felt strongly that useful application of Semantic Web to biomedicine would only occur if the technology was applied to and rooted in realistic use cases, and if the various task forces were encouraged to have their work interoperate within a common domain. Although medical research and practice generally depend on data sets covering genetics to clinical outcomes, research in and therapy development for the neurodegenerative disorders is a particularly striking illustration of the need for active, ongoing, synthesis of information, data, and interpretation from many sources and subdisciplines in biomedicine. For this reason, the HCLSIG is currently exploring use cases involving neurodegenerative diseases such as PD and AD. Next, we illustrate some of the issues with a scenario of a clinical researcher attempting to develop immunotherapies for AD.

### Alzheimer's disease immunotherapy scenario

A scientist working in a research hospital is pursuing immunization therapy for AD. A clinical trial of a vaccine made of synthetic Abeta1-42 ended prematurely a few years ago because 15 volunteers developed cerebral inflammation [47]. However, the field remains enthusiastic about new immunization strategies to reduce Abeta in early Alzheimer's, believed to be the culprit of AD [48], and to study the mechanism of action of Abeta immunization [49]. Important steps would be to identify the specific form of Abeta that is toxic to neurons and/or other elements critical to proper CNS function, and the mechanism of its toxicity.

The scientist uses her local scientific knowledge management system (*sci-know*) to search the Alzheimer Research Forum Web site and finds a recently published hypothesis (Abeta*56 Hypothesis) claiming a newly identified assembly of amyloid beta peptide, Abeta*56, causes memory impairment [4]. However, the hypothesis is based on claims only supported by experimental results from a transgenic mouse model. She wonders if Abeta*56 is found in actual AD patients, particularly in the early stages.

Based on the terms tagged to the hypothesis, that along with the original citation have been added to *sci-know*, the investigator constructs a search adding the concept *human *to the original query. The new query is run against PubMed and the hypothesis repository. Drawing on the ontology in the vicinity of the search terms to cluster the results, one research article comes to the forefront:

i. Using a novel, attomolar detection system, Amyloid-beta Derived Diffusable Ligands (ADDL) are elevated 8-fold on average (max 70-fold) in the cerebrospinal fluid of patients with AD [50].

The Alzforum AD Hypothesis knowledgebase shows (i) is cited as supportive evidence for the ***ADDL Hypothesis ***claiming *ADDL causes memory impairment*. Though the Abeta*56 hypothesis does not yet include a proposed mechanism for memory loss in the mouse model, the ***ADDL hypothesis ***includes a finding that ADDLs bind to human-derived cortical synaptic vesicles [51], and they inhibit hippocampal long-term potentiation (LTP) [52], a form of synaptic plasticity known to be critical for certain forms of learning and believed to be equally critical for memory storage [53,54]. Additional supporting evidence cited for this hypothesis notes Abeta alters A-type K+ channels involved in learning and memory, leading to altered neuronal firing properties as a prelude to cell death in Drosophila cholinergic neurons [55]. This provides a possible mechanistic explanation for the demonstrated learning disabilities, memory dysfunction, and neurodegeneration in transgenic Drosophila expressing human Abeta [56].

Are these model organism findings relevant to patients with AD? The researcher wonders whether A-type K+ channels are plausible therapeutic targets for treating patients diagnosed with AD. She asks:

"Show me the neuron types affected by early AD."

The *sci-know *system searches the Alzforum and comes up with several instances of neuronal cell types damaged in AD. These include BDNF neurons of the nucleus basalis of Meynert [57,58] and CA1 pyramidal neurons of the hippocampus [59]. Next, the researcher asks:

"Do BDNF neurons or CA1 pyramidal neurons have A-type K+ channels?"

"Are there other studies relating amyloid derived peptides to neocortical K+ channels?"

The application returns results from a neuropharmacological knowledgebase, BrainPharm. [60]. BrainPharm indicates CA1 pyramidal cells have A-type potassium channels. Interestingly, this finding carries the following annotation:

"Application of beta-amyloid [Abeta] to outside-out patches reduces the A-current; leading to increased dendritic calcium influx and loss of calcium homeostasis, potentially causing synaptic failure and initiating neuronal degenerative processes." [61].

Our researcher wonders whether the 56 kD form of Abeta is responsible for this effect and is led to a series of scientific questions she would like to address in her lab. Would the Tg2576 mouse model, the one in which Abeta*56 was reported to correlate with memory impairment, have a reduced A-current? Would blocking Abeta*56 with an antibody restore the A-current level? Our researcher types in one more query:

"Is there an antibody to Abeta*56 or ADDL?"

The application searches across a number of antibody resources and identifies one in another researcher's shared antibody database that even lists the e-mail address of the laboratory where she can obtain the antibody.

### Making data available in RDF and OWL

In our scenario, a number of queries are posed for a variety of types of biomedical knowledge. We query for specific types of neurons, the types of their associated ion channels, for the properties of amyloid derived peptides and their molecular interactions, for hypotheses and discussions about them, and for antibody reagents. Much, but not all, of this information is available in publicly accessible data sets. However, in order for them to be used on the Semantic Web, they need to be made accessible as RDF or OWL. The BioRDF group is exploring a number of methods for doing this. Among the data sets we have converted, and plan to make publicly available, are:

• **SenseLab. **The subset of *SenseLab *[62] that contains information about pathological mechanisms related to Alzheimer's Disease (*BrainPharm*) has been converted into RDF and the subset containing information about neuronal properties (*NeuronDB*) has been converted into OWL.

• **CoCoDat**. CoCoDat [63] is repository of quantitative experimental data on single neurons and neuronal microcircuitry A subset of information about ionic currents in different types of neurons has been converted into OWL.

• **Entrez Gene. **As described in [64], the Entrez Gene repository of gene-centered information was converted in its entirety to RDF.

• **PDSP Ki DB. **The *PDSP Ki Database *[65] is a repository of experimental results about receptor-ligand interactions and has a strong emphasis on neuroreceptors. It has been converted into OWL that conforms to an extended version of the established *BioPAX *[66] ontology for biomedical pathways.

• **BIND**. The *Biomolecular Interaction Network Database *(*BIND*) [67] is a large collection of molecular interactions, primarily protein-protein interactions. Like the PDSP KiDB, the OWL version of *BIND *is based on the *BioPAX *ontology.

• **Antibodies **– A collection of commercial antibody reagent data derived from the Alzforum Antibody Directory [68] and by crawling reagent vendor sites has been rendered in OWL.

In addition to the RDF and OWL data sets produced by the HCLSIG participants, there is a growing collection of RDF and OWL data sets that have been made available. Among these data sets are the OBO ontologies [69], Reactome [70], KEGG [71], NCI Metathesaurus [72], and UniProt [73].

Below we briefly discuss three approaches we have used to make data sets available in RDF.

#### CoCoDat

D2RQ [74] is used to provide access to CoCoDat. D2RQ is a declarative language to describe mappings between relational database schemas and either OWL or RDFS ontologies. The mappings allow RDF applications to access the contents of relational databases using Semantic Web query languages like SPARQL. Doing such a mapping requires us to choose how tables, columns, and values in the database map to URIs for classes, properties, instances, and data values. We illustrate some of these considerations by walking through a portion of the D2RQ document describing the mapping of CoCoDat's relational database form to RDF. In it, we see how rows in the *Neurons *table are mapped to instances, the column *ID_BrainRegion *is mapped to a property, and the string values of that column are mapped to URIs.

**@prefix d2rq: http://www.wiwiss.fu-berlin.de/suhl/bizer/D2RQ/0.1#**.

**@prefix: http://semweb.med.yale.edu/NeuroWeb/owl/cocodat#**.

**@prefix db1: http://semweb.med.yale.edu/NeuroWeb/db/cocodat#**.

The first task is to define the *namespace bindings *[75]. A namespace binding associates an abbreviation with a prefix used for a set of URIs. Following Semantic Web practice, all identifiers used in the mapping description are URIs. The mapping needs to use identifiers defined by D2RQ, identifiers we will generate for the RDF version of CoCoDat, and identifiers for parts of the relational database.

• "d2rq:" is the abbreviation for the namespace of identifiers used by D2RQ.

• "db1:" is the abbreviation for the namespace of identifiers of parts of the relational database.

• As identifiers should be globally unique, and the group undertaking the translation controls the domain 'semweb.med.yale.edu', the namespace for new identifiers in the RDF version of CoCoDat is based on that domain. This is chosen to be the *default namespace*, abbreviated as ":".

   **db1:CoCoDatDB rdf:type d2rq:Database; d2rq:odbcDSN "cocodat";**

Now the relational database where CoCoDat is stored is identified as "db1:CoCoDatDB" and defined by its connection via ODBC.

   **db1:RecordingNeuronSite rdf:type d2rq:ClassMap;**

   **d2rq:class :RecordingNeuronSite;**

   **d2rq:uriPattern ":RecordingNeuronSite-@@Neurons.ID@@";**

   **d2rq:dataStorage db1:CoCoDatDB.**

Following that, each row of the database table *Neurons *is mapped to an instance of the OWL class called *:RecordingNeuronSite*. The URI of each instance is constructed using the primary key of the table, *ID*. Therefore, the instance with the primary key 1 will have the URI "http://semweb.med.yale.edu/NeuroWeb/owl/cocodat#RecordingNeuronSite-1", abbreviated *:RecordingNeuronSite-1*.

   **db1:inBrainRegion rdf:type d2rq:ObjectPropertyBridge;**

   **d2rq:belongsToClassMap db1:RecordingNeuronSite;**

   **d2rq:property :inBrainRegion;**

   **d2rq:pattern "@@Neurons.ID_BrainRegion@@";**

   **d2rq:translateWith db1:BrainRegionTable.**

In this step, the *ID_BrainRegion *column in the Neuron table is mapped to the property *:inBrainRegion*. The values of that column are not to be used directly, instead undergoing a translation that is defined next.

   **db1:BrainRegionTable rdf:type d2rq:TranslationTable;**

   **d2rq:translation [d2rq:databaseValue "GM-Ctx_B"; d2rq:rdfValue :barrel-cortex;];**

   **d2rq:translation [d2rq:databaseValue "GM-Ctx_Gen"; d2rq:rdfValue :general-cortex;];**

   **d2rq:translation [d2rq:databaseValue "GM-Ctx_SeM"; d2rq:rdfValue :sensorimotor-cortex;];**

In this last step, we see a portion of the mapping of values from the ID_BrainRegion column. The string values in this column are meant to represent brain regions. Knowing that it is likely these values will need to be equated with terms from other ontologies, a decision is made to represent them as URIs. Later, one will be able to use owl:sameAs to equate these terms with others. With this mapping, the string "GM-Ctx_B" is translated into the URI "http://semweb.med.yale.edu/NeuroWeb/owl/cocodat#barrel-cortex".

The result of this mapping specification will be the creation of statements such as <:RecordingNeuronSite-1><:inBrainRegion><:barrel-cortex>, assuming the ID of the first row of the *Neurons *table is *1 *and the value in the *ID_BrainRegion *column is "GM-Ctx_B".

#### Entrez Gene

The XML version of Entrez Gene was transformed to RDF using XSLT [76]. The XML source is 50 GB and the generated RDF consists of 411 million triples. The Oracle Database 10g RDF Data Model was used to store and query the data. Although it would have been expedient to use XML element names directly as RDF properties, we instead mapped the element names to property names that were more descriptive and adhered better to accepted RDF style. For example, the element *Gene-track_geneid *was changed to the property *has_unique_geneid*. An authoritative URI naming scheme for NCBI resources does not exist, so the namespace "http://www.ncbi.nlm.nih.gov/dtd/NCBI_Entrezgene.dtd/" was created for use in this prototype.

**Antibodies. **The curation of information about antibody reagents is much less mature than that about genes and many other biological entities. Therefore, creation of this resource had a number of interesting problems. The most difficult challenge was how to associate antibodies with proteins. The query in our scenario depends on this association, yet the Alzforum directory and most commercial reagent vendors do not associate antibody targets with well known identifiers. Instead, they are listed by gene, protein, or molecule name. Our focus was on antibodies that react with proteins. Determining the referent of antibody names can be difficult because of the large number of gene and protein synonyms. This is further complicated because names can have variant spellings, antibodies can be non-specific, vendors can use idiosyncratic names, and protein names are often embedded in a product name. Our approach was to collect gene and protein synonyms from a variety of public databases – Entrez Gene, UniProt, OMIM [77], and Enzyme [78]. Sets of transformation rules (based on regular expressions) were applied to product listings to extract protein names, normalize common spelling variations, and recognize certain forms of lists. Finally, only unambiguous matches to names were considered reliable enough to use.

Understanding the provenance and terms of usage of data is important within science. We therefore created RDF using the FOAF [79] vocabulary to describe the Alzforum project, and used Dublin Core [80] properties to identify usage policies for the data. This RDF was linked to the newly compiled Alzforum antibody listing.

### Curating and navigating disease hypotheses, claims, and evidence

In our scenario, an essential part of the navigation that leads the scientist from desired therapy to molecular mechanism is based on relationships between hypotheses. Although much of what we represent in biomedical databases are experimental measurements or observations, the act of creating and consuming knowledge occurs in a complex web of activities and relationships. From this perspective, one way to view biomedical knowledge is as an incomplete network whose "growing edges" contain unresolved contradictions, i.e. varying interpretations of experimental data in relation to hypotheses.

A natural science focused ontology of AD might contain the relationship <NeurofibrillaryTangle><locatedIn><Neuron>, asserting a known fact. However, for active researchers in a field, many times the most interesting relationships are those that that are just emerging, i.e. they cannot yet be considered validated, and are often the subject of scientific controversy. Perhaps more than anywhere, the collection of these hypotheses, claims, and disputes characterizes the world of science and provides the raw material propelling experiments, grants, and publications. How, then, can we assist scientists in taking advantage of this class of knowledge?

SWAN (Semantic Web Applications in Neuromedicine), developed in part by members of the HCLSIG, is an application that focuses on enabling AD researchers to curate, organize, annotate, and relate scientific hypotheses, claims and evidence about the disease. The ultimate goal of this project is to create tools and resources to manage the evolving universe of data and information about AD, in such a way that researchers can easily comprehend their larger context ("what hypothesis does this support or contradict?"), compare and contrast hypotheses ("where do these two hypotheses agree and disagree?"), identify unanswered questions, and synthesize concepts and data into more comprehensive and useful hypotheses and treatment targets for this disease.

The application is oriented towards use by both the individual researchers and within the community. Therefore the application supports both secure personal workspaces as well as shared, public workspaces.

The 2005 pilot application was developed as a proof of concept for hypothesis management [81]. In SWAN, personal and public knowledgebases are structured as RDF triple stores manipulated by the Jena framework [82]. Content can be exported and shared peer-to-peer or via public knowledge servers. Neuroscientists and scientific editors have used the system. Knowledge in the workspaces has been integrated with data from SenseLab and other data sets using the Oracle RDF Data Model [83,84]. Development continues and initial deployment will be as part of the Alzheimer Research Forum Web site [85].

### Working with clinical guidelines

Much effort has been devoted to representing clinical guidelines and protocols in a machine-executable format [46]. The high cost of creating these frameworks and the specialized software needed to use them has hindered wide adoption of such systems. One challenge is that the encoded guidelines are not generally interoperable between systems, diluting what could be a combined effort to build this valuable resource. We observe that much of the technology needed to represent and execute such guidelines is available as part of the Semantic Web stack. Thus, we are experimenting with using Semantic Web technologies to implement such guidelines in order to show their effectiveness and to give feedback to developers on where additional capabilities are needed. Working within the Semantic Web would benefit this field for at least two reasons. First, the open standards for the technologies on which such systems can be built would encourage researchers and vendors to build systems that can interoperate. Second, it would speed development of such systems by making it easier for them to incorporate essential and current biomedical knowledge created by others, saving the cost of encoding that knowledge in each system that uses it.

Adaptability to changing conditions is an important requirement for making clinical recommendations. These changes take the form of a patient's condition progressing in potentially unpredictable ways, and new medical research and clinical trials that should be considered in addition to established guidelines.

Within ACPP we have modeled guidelines as directed graphs using RDF and OWL [86]. Within such a network, each node is a task. Depending on the granularity desired by clinical practices using the guideline, the task might be a process or a set of processes. Each process is designed to accomplish a clinical goal, such as acquiring knowledge via a diagnostic test and is associated with its expected outcome and a desired timeframe for that outcome. OWL is used to represent the ontology of clinical goals and outcomes following [87].

Each task has a *context *describing a set of sufficient conditions that make the process worthy of recommendation and safe to carry out. The context describes a mix of the patient's clinical and physical conditions, treatment status, and care setting. For example, it can make reference to states of prior or parallel processes, such as whether they were completed or aborted, and clinical settings such as a long term care centre, or an emergency room. These conditions are organized into *inclusion *and *exclusion *criteria. Inclusion criteria may be weighted and a minimum sum of weights of satisfied criteria is specified as a threshold above which a task can be recommended.

As an example, consider the treatment of dementia in AD patients. Prescription of cholinesterase inhibitors such as donepezil, rivastigmine, and galantamine are recommended based on evidence from clinical trials [88]. In our model, using OWL, *prescribingCholinesteraseInhibitors *is an instance of the *Process *class. An inclusion criterion would be a diagnosis of either AD Dementia, PD or Lewy Body Dementia (DLB). These diagnoses are represented as classes, and so the inclusion criterion can be represented as an OWL union of the classes. Exclusion criteria would be vomiting or other severe gastrointestinal disorders.

A clinical decision support system can recommend the next task in a patient-specific pathway based on rules. Although we have used OWL for evaluating rules using instance classification, the current standard is not expressive enough to use the weights and thresholds we assign to criteria in class definitions. To implement the following, we use Notation 3 [89] rules. All tasks are evaluated in the following way to see which are candidates for recommendation.

• Query the healthcare information network for all past and present patient conditions mentioned in the inclusion or exclusion criteria.

• If any exclusion criteria hold then discard the task.

• Collect the satisfied inclusion criteria.

• Add the weights assigned to each satisfied inclusion criteria.

• If the sum exceeds the threshold, the task may be recommended.

Regular re-evaluation during periods of patient stability and upon any change in medical condition allow us to adapt the treatment plan to the current medical situation.

This approach to representing guidelines is also well suited to the incorporation of new knowledge. Each guideline would be available as an individual RDF or OWL document uniquely identified by its URI. Trusted sources would be identified that maintain up-to-date guidelines and protocols. Analogous to the *contexts *of tasks, each guideline or clinical trial would be associated with its own inclusion and exclusion criteria that would qualify the whole body of knowledge, i.e. all tasks described in the guideline. With this approach, the same form of rules used to identify relevant tasks would be used to identify relevant guidelines [90]. The tasks from all relevant guidelines and protocols would then be evaluated to determine the set of recommendations. By applying this method, if a patient has multiple clinical conditions, all relevant guidelines can be utilized to ensure doctors have appropriate information to ensure the best possible treatment for their patients.

## Discussion

### Data integration

There is a tacit assumption within the Semantic Web community that every data set and ontology will interoperate. The reality is that different conceptualizations and representations of the same data can exist. While the architecture and basic tools of the Semantic Web remove a set of previous roadblocks to data integration, positive progress towards it requires study, experimentation, and at-scale efforts that exercise proposed solutions.

To date, we have primarily focused on building prototypes that have functioned independently. Much of the RDF and OWL that has been generated mirrors the structure of the original data sets. Such translations are more syntactic than semantic. Even so, the common syntax enables an easier creation of cross-domain queries. As an example, in [83] the RDF translation of BrainPharm and SWAN's publication, data in RDF format were loaded into a single RDF store. Having both data sets available simultaneously allowed interesting new queries. For example, one could retrieve commentary by Alzforum members on articles that discussed drugs for which BrainPharm had models about cellular mechanism of action. This type of query succeeds because the two data sets being integrated do not, for the most part, discuss the same type of entity.

In order to integrate data sets, one of two things must happen: either terms for entities and relationships must be shared between the data sets (the data sets must be built using a shared ontology) or concordances must be available that relate terms in one data set to those in another.

Even when the ontology is shared, there is no guarantee that integration will be successful. Consider the BioPAX exchange format, an OWL-based ontology that provides a common framework for the many data sources that are repositories of information on cellular pathways. Despite the common ontology, it remains difficult to query an aggregation of different sources of BioPAX formatted data, e.g., for interactions related to the glucose metabolism pathway. This is because the terms shared among the data sources (the ones defined in the BioPAX standard) do not cover the scientific domain adequately to support such a query.

Building such ontologies is hard. The ontologies task force has therefore started focusing on identifying available knowledge resources (e.g., thesauri, terminologies, ontologies) that cover the basic biomedical entities and relations required to formally represent well defined scenarios like the one we present above.

While concepts in evolving areas of research may be incomplete, unclear, in transition or under dispute, there are many important entities and relations upon which most biomedical researchers and clinicians will agree. Mitochondria are found inside viable eukaryotic cells, voluntary movement in humans requires functional innervation of skeletal muscles, etc.

Our first goal is to construct a skeleton ontology specifying the required high-level biomedical domains, and, then to determine which public resources provide the required domain entities and relations along with clear prose definitions of them. These textual definitions are essential to guide curators and translators of data sets towards consistent usage of terms. Where definitions that we need do not exist in public resources, we will attempt to define the terms and work with others in the biomedical ontology community to refine and formalize them.

For example, an important term in our scenario is *Ion Channel*. In order to pose a query about ion channels and retrieve information about *A-type K+ channels*, we need to ensure that the definition is clear enough that competent informaticians who are not necessarily domain experts have enough hints to gather sufficient information to realize that a *K+ channel *is an ion channel.

It is important that the same attention that is given to identifying and defining classes is also given to defining relationships (properties) [91]. There are fewer definitions for such relationships, in public resources, than for classes. For example, in order to record details of the hypotheses in our scenario, we need to define the relationship between Abeta and development of symptoms of AD. Therefore we might define "isAPeptideContributingCauseOf" to be "a potentially causal relationship between peptides such as Abeta1-42, Abeta*56 and a disease such as AD or a clinical condition such as Memory Impairment". The definition notes the type of subject (peptide) and object (clinical condition or disease) of the property that will formally link, as domain and range of the property, and then to classes in our ontology. This definition will serve as our input to other communities working in this domain – for example when we participate in an upcoming workshop on clinical trial ontologies organized by the National Center for Biomedical Ontology (NCBO) [92].

### Current technical limitations of semantic web

Semantic Web technologies are young. Gaps in standards and implementations still exist and adoption is limited by typical problems with early technology, such as the need for a critical mass of practitioners and installed base, and growing pains as the technology is scaled up. Some issues that have affected the work of the HCLSIG are:

#### Scarcity of semantically annotated information sources

Although we have listed a number of public sources of data that are available in RDF, most common sources of data for bioinformatics are not currently in a RDF or OWL. However, mapping tools such as D2RQ should lower the barrier to making these data sets available.

#### Performance and scalability

RDF and OWL stores are slower than optimized relational databases, but are improving steadily [93]. However, logical reasoning over large or complex ontologies remains a problem.

#### Representation of evidence and data provenance

It is often important to know where knowledge has come from and how it has been processed. It is also useful to know who believes something and why. However, there is no standard way of expressing such information about a statement or collection of RDF statements. *Named graphs *[94] may solve many of these problems and are already being employed in projects such as myGrid [95] to trace data provenance. However, they are not a standard and, therefore, are not widely supported by Semantic Web tools.

#### Lack of a standard rule language

Although there are technologies that enable the use of rules, there is no standard rule language. This makes it impossible to write sets of rules that can be used in different implementations, limiting the reach of the ACPP group's vision of distributed clinical guidelines encoded as rules. We note, however, that the W3C Rule Interchange Format Working Group [96] is currently working to solve this problem.

### Cross-community interactions

There is an emerging consensus in the bioinformatics community at large for the need to formalize and share data annotation semantics. This is championed by such institutions as the UK e-Science project myGrid [97], the Bio-Health Informatics Group [98] at the University of Manchester, U.K., the NIH-funded National Center for Biomedical Ontology [42,99], and the growing Open Biomedical Ontologies (OBO) Foundry [100].

The Semantic Web and biomedical communities need to further coordinate efforts in areas critical to translational research, namely:

• Formalizing the semantics of the elements of health care information systems, such as medical records, as well as clinical decision making, such as disease and symptoms.

• Making scientific publishing more effective at supporting research communities by finding ways to systematically capture research results and make them available on the Semantic Web.

• Engaging systems biology researchers as "early adopters" of Semantic Web technologies, and as a resource for driving use cases.

• Working with natural language processing researchers to enhance their algorithms with biomedical ontologies, and to target their output to use terms from established ontologies.

• Working with the U.S. National Library of Medicine (NLM) to find appropriate ways to translate their extensive vocabularies and knowledge resources into RDF for effective use on the Semantic Web.

As discussed in [101], tensions have occurred between the Semantic Web communities and other communities like the XML and database communities, as some people believe that the technologies being advocated by these communities cannot coexist with each other. One way to ease such tensions is for the Semantic Web community to develop a complementary rather than competitive relationship with these communities. The Semantic Web should be perceived as a complement instead of a replacement to existing technologies. For example, RDF/OWL can be serialized as XML, and can be used to provide a richer semantic layer for use with other XML technologies. The developers of triple stores and RDF query languages have been greatly inspired by the theoretical and practical work done by the database community. Providers of valuable knowledge such as curators of biological pathways would be more willing to make their data accessible to the Semantic Web community if they did not need to abandon their own formats. For example, converters can be provided for translating BioPAX into other pathway data formats so that tools that were built based on these formats can still be used. At the same time, additional tools can be developed to exploit the new features (e.g., reasoning) enabled by representing BioPAX in OWL.

### Education and incentives

The vision of a Semantic Web accelerating biomedical research crucially depends on the holder of scientific and clinical data making that data available in a reusable form. Often the effort that goes into preparing and serving this data will not directly benefit the provider. Instead, researchers are measured for producing scientific discoveries and writing about them, doctors for helping sick patients, and pharmaceutical companies for producing safe, effective drugs. There are also privacy risks involved with sharing personal information. Valuable patient data can only be acquired with appropriate consent and with sensitivity to those privacy issues. It is an open question of how to structure incentives to make these holders of valuable information consider the effort to be in their best interest.

If the research community decided today that it was motivated to publish data semantically, we do not yet have adequate numbers of skilled knowledge workers. Data modelling even without the intention of interoperating is a hard-learned skill, and the challenge is substantially magnified when the intention is to share information for unforeseen uses. We need to establish and populate a new discipline, a mix of interdisciplinary skills that include solid understanding of biomedicine, computer science, philosophy and the social anthropology of science and computing.

## Conclusion

We have discussed the potential of the Semantic Web to facilitate translational research. Although Semantic Web technologies are still evolving, there are already existing standards, technologies, and tools that can be practically applied to a wide range of biomedical use cases. There are challenges to the widespread adoption of the Semantic Web in the health care and life sciences industries. Some parts of the technology are still in development and are untested at large scales. Informaticians need training and support to be able to understand and work with these new technologies. Incentives need to be provided to encourage appropriate representation of important research results on the Web.

By grounding the development and application of this technology in real concerns and use cases of the biomedical community, and enabling close interaction between informaticians, researchers, and clinicians, and the W3C standards development community, the W3C HCLSIG is providing a rich collaborative environment within which to start resolving these issues. The potential of interoperable knowledge sources for biomedicine, at the scale of the World Wide Web, certainly merits continued attention.

## Competing interests

The authors declare that they have no competing interests.

## Authors' contributions

KC initiated and orchestrated the effort of writing the paper. All authors have contributed to the manuscript and participated in the discussions at the face-to-face meetings, teleconferences and on e-mail. IH, EN, and TH helped facilitate forums for discussing the paper. JK, TC, EW, GW, and WB developed the AD immunotherapy scenario. AR edited the manuscript, with help from TC, WB, KC, JR, SS, and SM.
